# Supplementary material for: Structural and dynamic insights into agonist recognition and function of the thromboxane A2 receptor
Source: Nat Commun. 2026 Feb 23;17:3071. doi: 10.1038/s41467-026-69844-9 (PMC13039517; doi:10.1038/s41467-026-69844-9)
Supplement: Supplementary file 1 — Supplementary Information [file 41467_2026_69844_MOESM1_ESM.pdf]

Supplementary Information

Structural and Dynamic Insights into Agonist Recognition by and Function of the Thromboxane A_2 Receptor: Implications for Disease-Causing Mutations

Pawel Krawinski[1†], Donna Matzov[2†], Aoife Ryder[1], Kanhaya Lal[3], Dmitry S. Karlov[3], Georges Chalhoub[4,5], Eamon P. Mulvaney[6], B. Therese Kinsella[6], Peter J. McCormick[4,5], Martin Caffrey[1*], Irina G. Tikhonova[3*], Moran Shalev-Benami[2*]

[1]School of Medicine and School of Biochemistry and Immunology, Trinity College Dublin, Dublin D02 R590, Ireland. [2]Department of Chemical and Structural Biology, Weizmann Institute of Science, Rehovot 7610001, Israel. [3]School of Pharmacy, Queen's University Belfast, Belfast BT9 7BL, UK. [4]Department of Pharmacology and Therapeutics, University of Liverpool, Institute of Systems, Molecular and Integrative Biology, Sherrington Building, Ashton Street, Liverpool L69 3GE, UK. [5]Centre for Endocrinology, William Harvey Research Institute, Barts and the London School of Medicine, Queen Mary, University of London, London, UK. [6]ATXA Therapeutics Limited, UCD Conway Institute of Biomolecular and Biomedical Research, University College Dublin, Belfield, Dublin 4, Ireland.

***Corresponding authors:** martin.caffrey@tcd.ie; i.tikhonova@qub.ac.uk; moransb@weizmann.ac.il

Supplementary Discussion

Supplementary Figures 1-15

Supplementary Tables 1-3

Supplementary References

Supplementary Discussion

TM1 involvement in the mechanism of receptor activation

In the MD simulation models of the active, agonist-bound states of the thromboxane (TX) A_2 receptor (TP), G38$^{1.46}$ forms a hydrogen bond network with N42$^{1.50}$ and Q301$^{7.46}$. N42$^{1.50}$ links TM1 to TM7 by using its amide group to hydrogen bond to the backbone carbonyls of both G38$^{1.46}$ and Q301$^{7.46}$. These interactions likely hold TM1 in the closed conformation. Given that the changes in TM1 conformation possibly happen as a result of agonist binding, the role of the movement of this helix in receptor activation was investigated by BRET assays of the G38$^{1.46}$A, G38$^{1.46}$V, N42$^{1.50}$S, and P28$^{1.36}$C/A90$^{2.66}$C mutants (Supplementary Fig. 4). The G38$^{1.46}$A and G38$^{1.46}$V mutants were unable to activate G_q suggesting that replacing glycine reduces helix flexibility and its ability to act as a hinge point. The N42$^{1.50}$S disease-related mutant negatively impacts the receptor likely due to the loss of hydrogen bonding between G38$^{1.46}$ and Q301$^{7.46}$ in the active TP (a serine is unable to hydrogen bond to both of these residues), and as already discussed, N42$^{1.50}$ is directly involved in the activation mechanism. A rationally designed disulphide bridge between TM1 and TM2 in the P28$^{1.36}$C/A90$^{2.66}$C double mutant, aimed at preventing TM1 movement, resulted in reduced agonist efficacy. These mutagenesis data strongly suggest that TM1 movement is necessary for receptor activation.

Voltage-dependent modulation

Voltage-dependent modulation is often neglected as a means for regulating receptor function. Changes in polarization state happen in most cells including platelets and not just those of excitable tissue such as neurons, and cardiac and smooth muscle. Of the receptors investigated in a recent study[1], the TP proved to be particularly sensitive. R295$^{7.40}$, which plays a key role in binding agonists and antagonists, was considered as a possible sensor. Given that R295$^{7.40}$ bears a permanent positive charge it makes sense that it might be involved in sensing changes in membrane potential and in relaying the information across the membrane for downstream signalling. Cryogenic electron microscopy (cryo-EM) studies of membrane-integral ion channels in an applied electric field suggest[2] that the investigation of voltage-dependent modulation of GPCR function is likely to become an important sub-discipline within the field[3].

MD Simulations

We performed molecular dynamics (MD) simulations of our cryo-EM structures of the TP in the presence and absence of the agonists U46619 and I-BOP. In addition, endogenous thromboxane, TXA_2 was docked to the TP and their corresponding complexes were simulated. Agonist complexes were simulated in the presence and absence of the G_q heterotrimer. We also simulated structures of the TP with and without the antagonists, ramatroban and daltroban, embedded in a hydrated lipid bilayer. The goal was to compare the stability, dynamics, and interactions within the different complexes. The lipid composition used in simulations matched the natural glycerophospholipid, sphingolipid, and cholesterol composition of platelets. The root mean square deviation (RMSD) and fluctuation (RMSF) of the Cα atoms were analysed for the entire receptor and its transmembrane domain. The RMSD and RMSF were also calculated for the non-hydrogen atoms of the ligand in the receptor-ligand complexes. These analyses showed that the complexes were stable and experienced only minor fluctuations (Supplementary Table 2). A reliability and reproducibility checklist for our MD simulations is provided in Supplementary Table 3.

Ligand interactions at the binding site

By incorporating water molecules and molecular dynamics, we found evidence of additional polar interactions between I-BOP/U46619/TXA_2 and the TP compared with the static cryo-EM structures. Specifically, the agonist carboxyl group forms a hydrogen bond network with $H89^{2.65}$, $S181^{ECL2}$, and $R295^{7.40}$ facilitated by bridging waters (Supplementary Figs. 6 and 7). Interestingly, ligand binding is more stable when $H89^{2.65}$ is protonated (Supplementary Table 2). To evaluate the protonation status of $H89^{2.65}$, we estimated the pK_a of $H89^{2.65}$ by calculating titration curves from constant pH simulations of ligand-bound and empty forms of TP. We found that the presence of the ligand shifts pK_a to more basic values, resulting in the ligand-bound forms being fully protonated at physiological pH (pH 7.4; Supplementary Fig. 5).

Additionally, water-mediated interactions were observed between the bicyclic ring oxygen and $T298^{7.43}$ (Supplementary Figs. 6 and 7). The agonist hydroxyl group donated a hydrogen bond to the carbonyl oxygen of $Q301^{7.46}$ side chain (Supplementary Fig. 6), which formed early in, and remained stable throughout, the MD simulations (Supplementary Fig. 6b).

The antagonist carboxyl group also engages in hydrogen bonding with H89$^{2.65}$, S181^{ECL2}, and R295$^{7.40}$ (Supplementary Fig. 6). However, the sulfonyl group of the antagonists acts as a hydrogen bond acceptor interacting strongly with the Q301$^{7.46}$ amide instead of behaving as a hydrogen bond donor like the hydroxyl group of the agonists (Supplementary Fig. 6b). The antagonist's amide forms a strong hydrogen bond with T81$^{2.57}$ (Supplementary Fig. 6b).

Analysis of the average ligand-residue interaction energies revealed other key binding interactions for the agonists (Supplementary Fig. 14). In addition, hydrogen bond interactions occurred between the ligand and the backbone of A31$^{1.39}$ and F30$^{1.38}$ and the side chains of S27$^{1.35}$, P179^{ECL2}, and W182^{ECL2} further stabilising the carboxyl group of the ligand. Strong van der Waals interactions were observed between the agonists and A31$^{1.39}$, L78$^{2.54}$, T81$^{2.57}$, V85$^{2.61}$, M112$^{3.32}$, W182^{ECL2}, L294$^{7.39}$, and T298$^{7.43}$ over the course of the MD simulations.

Activation mechanism

In the antagonist-bound receptors, our simulations agree with the crystal structures showing Q301$^{7.46}$ hydrogen bonding with W258$^{6.48}$ to maintain the inactive state (Supplementary Fig. 6b). Upon agonist binding, Q301$^{7.46}$ loses this bond and, instead, interacts with the agonist hydroxyl group (Supplementary Fig. 6b). This enables W258$^{6.48}$ to form a hydrogen bond with N300$^{7.45}$ (Supplementary Fig. 6), leading to TM7 rotation as seen in the simulations and cryo-EM structures (Supplementary Fig. 15). Thus, W258$^{6.48}$ adopts inactive or active conformations by interacting with Q301$^{7.46}$ or with N300$^{7.45}$, respectively (Supplementary Fig. 6).

We simulated D74$^{2.50}$ in the protonated state, as it is predicted to be protonated during activation of other GPCRs. This protonated D74$^{2.50}$ forms a hydrogen bond with D304$^{7.49}$ of the D$^{7.49}$P$^{7.50}$xxY$^{7.53}$ motif, bringing the Q301$^{7.46}$ backbone close to the amide side chain of N42$^{1.50}$ to form a hydrogen bond (Supplementary Fig. 6b). This has the effect of drawing TM7 closer to TM1, TM2, and TM3, stabilizing the hydrogen bond between G123$^{3.43}$ and Y308$^{7.53}$ of the D$^{7.49}$P$^{7.50}$xxY$^{7.53}$ motif indicative of activation (Supplementary Fig. 6b). Importantly, we do not observe this network involving D74$^{2.50}$ in antagonist-bound simulations, where TM7 remains dynamic (Supplementary Fig. 15).

The tighter clustering between TM1, TM2, TM3, and TM7 upon activation displaces internal water molecules towards TM4, TM5, and TM6 (Supplementary Fig. 6). This likely facilitates

the known outward movement of TM5 and TM6 during activation. The simulations show TM6 exhibits increased mobility and outward displacement with the agonist bound (Supplementary Fig. 15). Additionally, the R130$^{3.50}$ of the E$^{3.49}$R$^{3.50}$Y$^{3.51}$ motif shifts towards an active conformation, moving away from E129$^{3.49}$ especially when the G_q heterotrimer is present (Supplementary Fig. 6b).

Ligand binding

Since the extracellular portion of the TP is occluded by ECL2, ECL3 and the N-terminus, we used random accelerated MD (RAMD) to explore dissociation pathways for agonists and antagonists. In RAMD, a random force is applied to the ligand centre of mass in various directions to generate a dissociation trajectory. The bulkier and more rigid antagonists, ramatroban and daltroban, primarily exited through the gap between the extra-cytoplasmic halves of TM1 and TM7 towards the lipid bilayer (96% and 84% of the trajectories) (Supplementary Fig. 10). In contrast, the more flexible agonists, U46619 and I-BOP, showed two main bilayer escape routes, namely the TM1/TM7 gap (40% and 32%) and the TM1/TM2 gap (50% and 46%). With much reduced frequency, the agonists also exited extracellularly between TM1, TM2, TM3, and ECL2 (6% and 13%), and to the bilayer through the TM2/TM3 gap (4% and 9%) (Supplementary Fig. 10). The high flexibility of TM1 seen in conventional simulations explains its featured role.

Supplementary Figures

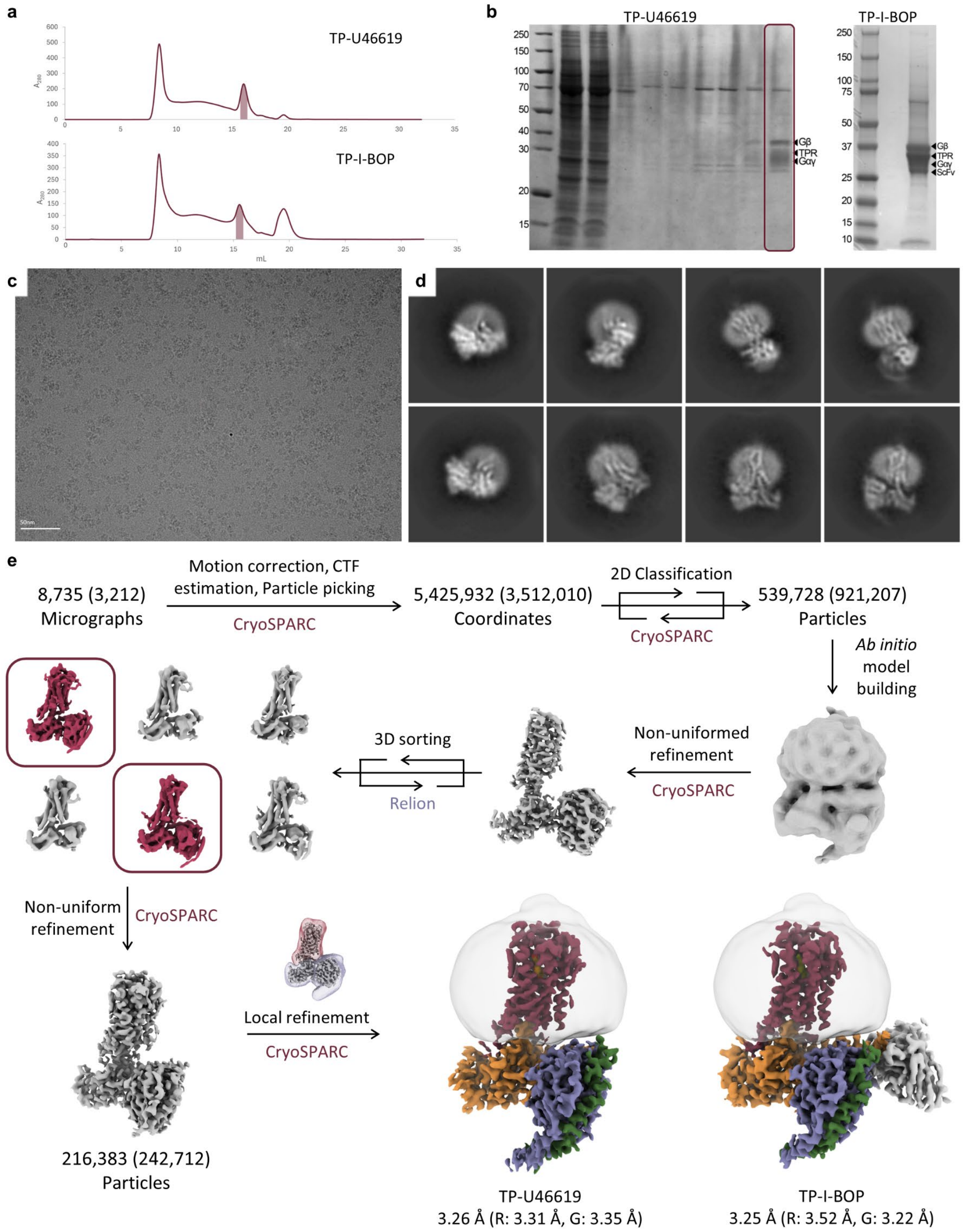

Supplementary Fig. 1 | Protein purification and cryo-EM flowchart. a Size exclusion chromatography (SEC) profile of the TP complex purified with the agonists U46619 (top) and I-BOP (bottom). Fractions corresponding to the monomer peak (dark red) were combined and used for the cryo-EM studies. **b** Coomassie-stained SDS-PAGE indicating the presence of the receptor and the G_q heterotrimer in the samples used for the structural studies. Data for TP-U46619 is presented to the left. The relevant lane containing the SEC fraction used for the EM studies is boxed with a red line. The TP-I-BOP complex (presented to the right) was prepared in the presence of scFv16. The band corresponding to scFv16 is labelled (ScFv). **c** Representative cryo-EM micrograph of the TP-I-BOP complex. **d** Representative reference-free 2D class averages. The diameter of the circular mask is 20 nm. **e** Processing flowchart of the cryo-EM data, including particle selection, 2D and 3D classifications, particle sorting, masking, and final map reconstruction. Data processing was done using a combination of CryoSPARC 4.2.1[4,5] and Relion 4.0[6,7]. The software used for each step is indicated in the flowchart.

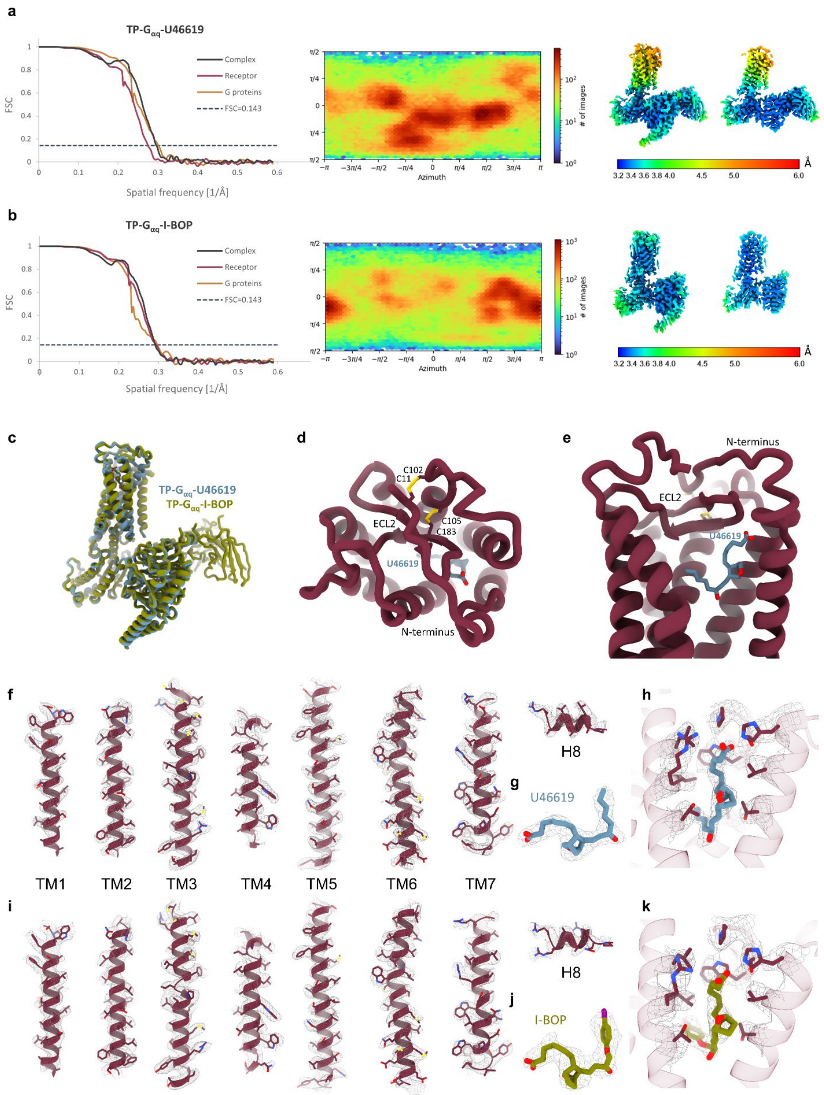

Supplementary Fig. 2 | EM map, model quality and structural features of the activated TP. a-b *Left:* Gold standard Fourier shell correlation (FSC) curves of half maps that were individually calculated for each reconstitution. The map calculated for the entire complex is in black. The maps calculated for the receptor alone and for the G_q heterotrimer alone are shown in red and gold, respectively. Resolutions for the TP-U46619 complex, receptor alone, and G_q heterotrimer alone are 3.26 Å, 3.31 Å and 3.35 Å, respectively (**a**). Resolutions for the TP-I-BOP complex, receptor alone, and G_q heterotrimer alone are 3.25 Å, 3.52 Å and 3.22 Å, respectively (**b**). Resolutions were calculated at 0.143 FSC. *Middle:* Angular distribution of particles for the final 3D reconstructions. *Right:* EM density map coloured by local resolution (calculated by Phenix). Colour represents the resolution in Å units. Side view (left) and cut through (right) of the map are shown. The map contour level is 0.3 and 0.25 for TP-U46619 (**a**) and TP-I-BOP (**b**), respectively. **c** Superposition of the two active structures with TP-U46619 (blue) and TP-I-BOP (green) indicating that the models are very similar with an all-atom RMSD value of 0.5 Å. **d-e** Two unique disulfide bonds are formed between C11$^{N\text{-}term}$ and C102$^{3.22}$, and between C105$^{3.25}$ and C183^{ECL2}, that create a rigid 2-layer lid in TP-U46619 that limits access of ligands to the orthosteric pocket from the extracellular milieu. Top (**d**) and side (**e**) views show disulfide positions and highlights the lid composed of ECL2 and the N-terminus. **f-h** Snapshots of model in density of the U46619-bound structure for TM1-7 and H8 (**f**), the ligand U46619 (**g**), and the binding pocket surface (**h**). The map contour level is 0.2 for the TMs and ligand, and 0.3 for the pocket representation. **i-k** Snapshots of model in density of the I-BOP bound structure for TM1-7 and H8 (**i**), I-BOP (**j**) and the binding pocket surface (**k**). The map contour level is 0.15 for the TMs and ligand, and 0.2 for the pocket representation.

a ECL2

**TP**	P	G	S	W	C	F	L
**DP1**	P	G	T	W	C	F	I
**DP2**	R	I	M	-	C	Y	Y
**EP1**	P	G	T	W	C	F	I
**EP2**	P	G	T	W	C	F	I
**EP3**	P	G	T	W	C	F	I
**EP4**	P	D	T	W	C	F	I
**FP**	S	R	T	W	C	F	Y
**IP**	P	G	S	W	C	F	L

b α-chain subpocket

**TP**	T	H	M	L	L	R	T
**DP1**	L	L	M	L	L	R	S
**DP2**	L	Y	F	P	T	S	F
**EP1**	P	Y	M	F	V	R	S
**EP2**	I	Y	M	L	L	R	S
**EP3**	T	Y	M	L	V	R	S
**EP4**	V	Y	L	L	I	R	S
**FP**	N	Y	M	L	L	R	T
**IP**	L	Y	M	L	F	R	A
	2.57	2.65	3.32	7.36	7.39	7.40	7.43

c Ring subpocket

**TP**	A	F	C	L	T	G	V
**DP1**	G	L	F	K	L	S	V
**DP2**	H	A	S	S	L	P	T
**EP1**	P	S	M	H	P	G	V
**EP2**	S	M	F	T	I	S	V
**EP3**	P	M	L	Q	T	T	V
**EP4**	P	M	F	T	V	S	T
**FP**	S	F	M	H	N	G	A
**IP**	S	M	F	T	L	S	V
	1.39	1.42	1.43	2.54	2.57	2.58	2.61

d ω-chain subpocket

**TP**	F	G	F	W	L	Q
**DP1**	F	G	S	S	V	S
**DP2**	N	M	K	W	Y	S
**EP1**	F	G	S	W	M	Q
**EP2**	F	S	T	S	F	S
**EP3**	F	G	F	W	L	Q
**EP4**	F	S	Y	S	L	P
**FP**	S	G	F	W	F	Q
**IP**	F	G	G	S	L	P
	3.35	3.36	5.43	6.48	6.51	7.46

Supplementary Fig. 3 | Sequence alignment of the TP with other prostanoid receptors. a The ECL2 of prostanoid receptors consists of a highly conserved PGTWCFI motif (consensus sequence highlighted in different colours) that forms the ceiling of the binding pocket. **b-d** Selected residues forming the α-chain (**b**), ring (**c**), and ω-chain (**d**) subpockets of the TP and their alignment with other prostanoid receptors. The Ballesteros-Weinstein residue numbers are shown below each column. In (**a**), the conserved $Y^{2.65}$ and $R^{7.40}$ residues that interact with the carboxylic acid group of endogenous prostanoid ligands are coloured purple and blue, respectively. The TP and DP1 are exceptions. The TP has a histidine (red) at position 2.65. Residues at positions 3.32 and 7.39 (cream) are all apolar and similar in size, playing a role in maintaining the correct geometry of the ligands. Polar and apolar residues in (**c**) are coloured light blue and cream, respectively, showing that the ring subpocket of TP is more apolar than the ring subpockets of other prostanoid GPCRs. $W^{6.48}$ is coloured navy in (**d**). The residue at position 3.36 often interacts with $W^{6.48}$ in other class A GPCRs, but the $G^{3.36}$ (green) in most prostanoid receptors is unable to do so. Polar Q and S residues (orange and yellow, respectively) are found at position 7.46. In the TP, a conservative mutation of $Q^{7.46}$ to $S^{7.46}$ did not abolish receptor activity.

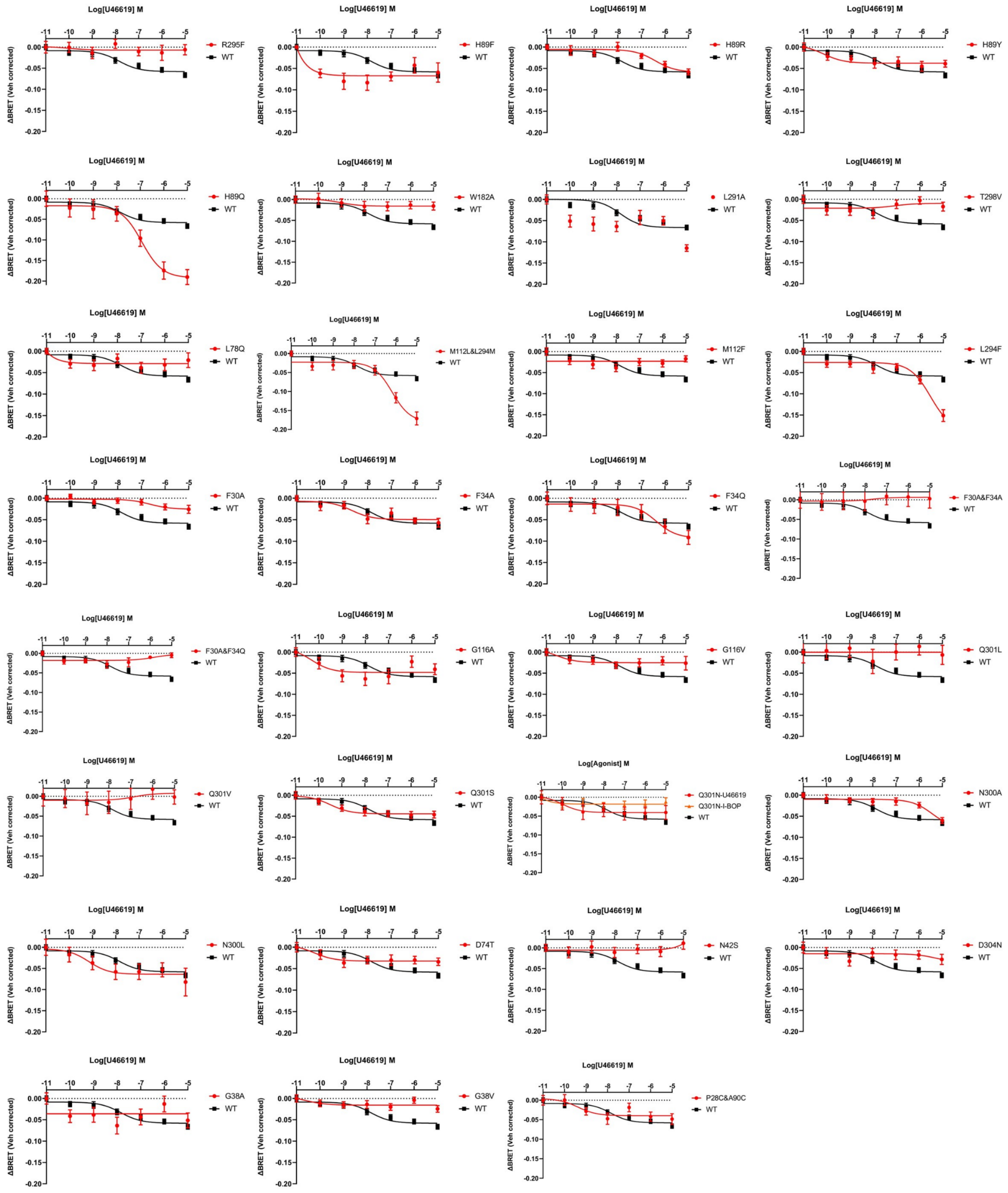

Supplementary Fig. 4 | BRET signalling assays of TP mutants. Dose-response curves of TP mutants following stimulation with U46619 and/or I-BOP (final concentration range of 10^{-11} – 10^{-5} M). Data are presented as the mean ± SEM of 3 independent experiments (n=3) performed in technical triplicate.

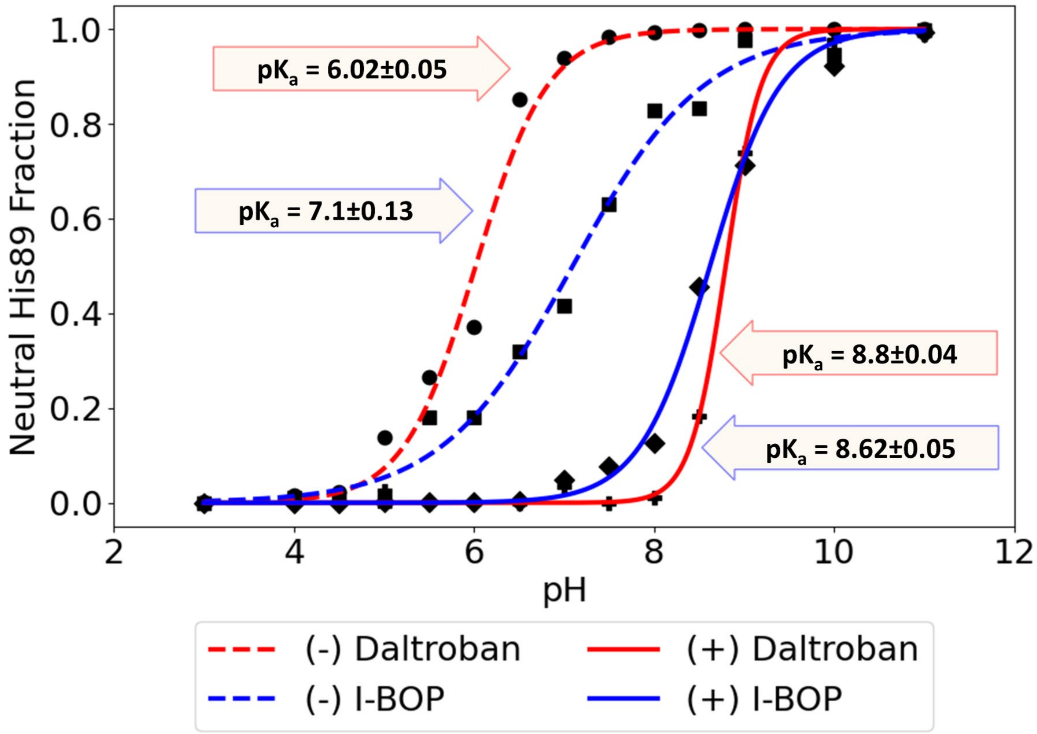

Supplementary Fig. 5 | pH titration curves and estimated pK_a values for H89$^{2.65}$ computed from constant pH simulations of the TP with or without bound antagonist (daltroban) or agonist (I-BOP). Ligand binding produces a shift in pK_a to more basic values making the imidazole of H89$^{2.65}$ in the ligand-bound receptor fully protonated (positively charged) at physiological pH.

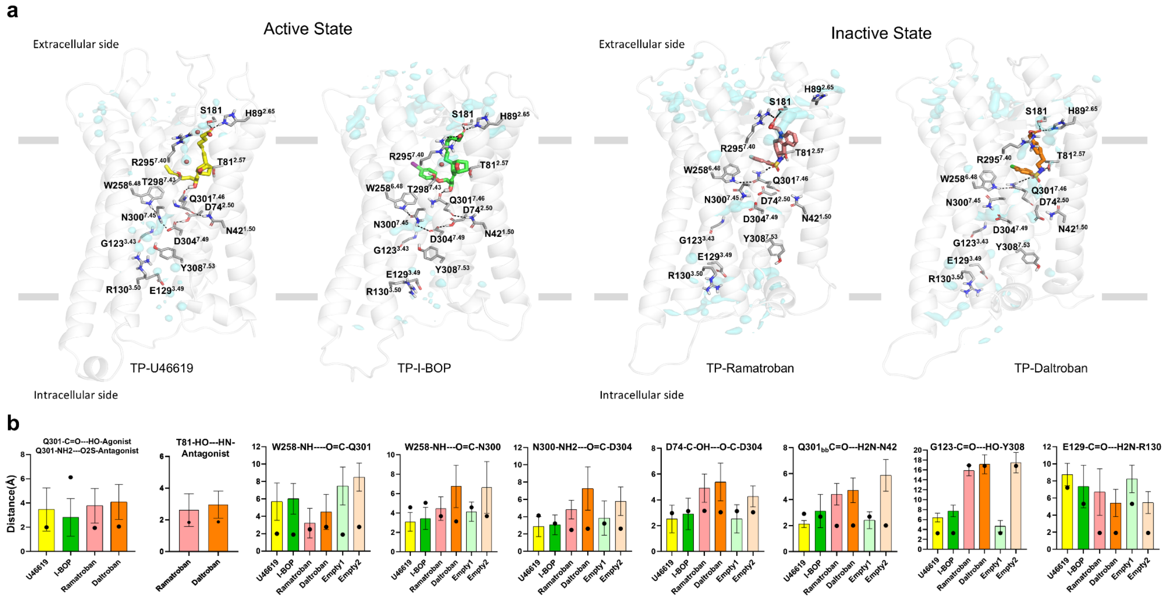

Supplementary Fig. 6 | MD simulations reveal the distinct hydrogen bond networks that agonists and antagonists form with the TP. a Representative simulation snapshots of the agonists, U46619 (yellow) and I-BOP (green), and antagonists, ramatroban (pink) and daltroban (orange), bound to the TP. Key residues involved in receptor-ligand interactions and receptor activation are shown. Black dashes indicate hydrogen bonds, cyan surfaces show internal water molecules. **b** Dynamics of key hydrogen bonding interactions in agonist- and antagonist-bound TP complexes. Mean distances between atoms involved in hydrogen bonding during MD simulations (≥1 μs, n=3) are shown. Simulations were initiated from U46619- or I-BOP-bound (agonists, yellow/green), ramtroban- or daltroban-bound (antagonists, pink/orange) structures, and empty receptors from U46619- and daltroban-bound complexes (light green/orange). Black dots represent distances in cryo-EM structures. Key findings include: stable hydrogen bonds (donor-acceptor distance <3.5 Å) between Q301$^{7.46}$(C=O)-agonist(OH) and Q301$^{7.46}$(NH_2)-antagonist(SO_2); stable T81$^{2.57}$-antagonist hydrogen bond; W258$^{6.48}$ hydrogen bond switch between Q301$^{7.46}$ (antagonist-bound) and N300$^{7.45}$ (agonist-bound); agonist-specific hydrogen bonds (N300$^{7.45}$-D304$^{7.49}$, D74$^{2.50}$-D304$^{7.49}$, Q301$^{7.46}$-N42$^{1.50}$, G123$^{3.43}$-Y308$^{7.53}$); and E129$^{3.49}$-R130$^{3.50}$ ionic lock of the E$^{3.49}$R$^{3.50}$Y$^{3.51}$ motif breaking (donor-acceptor distance >5 Å) in agonist-bound but not in antagonist-bound complexes. These interactions highlight distinct conformational changes associated with agonist and antagonist binding, providing insights into TP activation mechanisms.

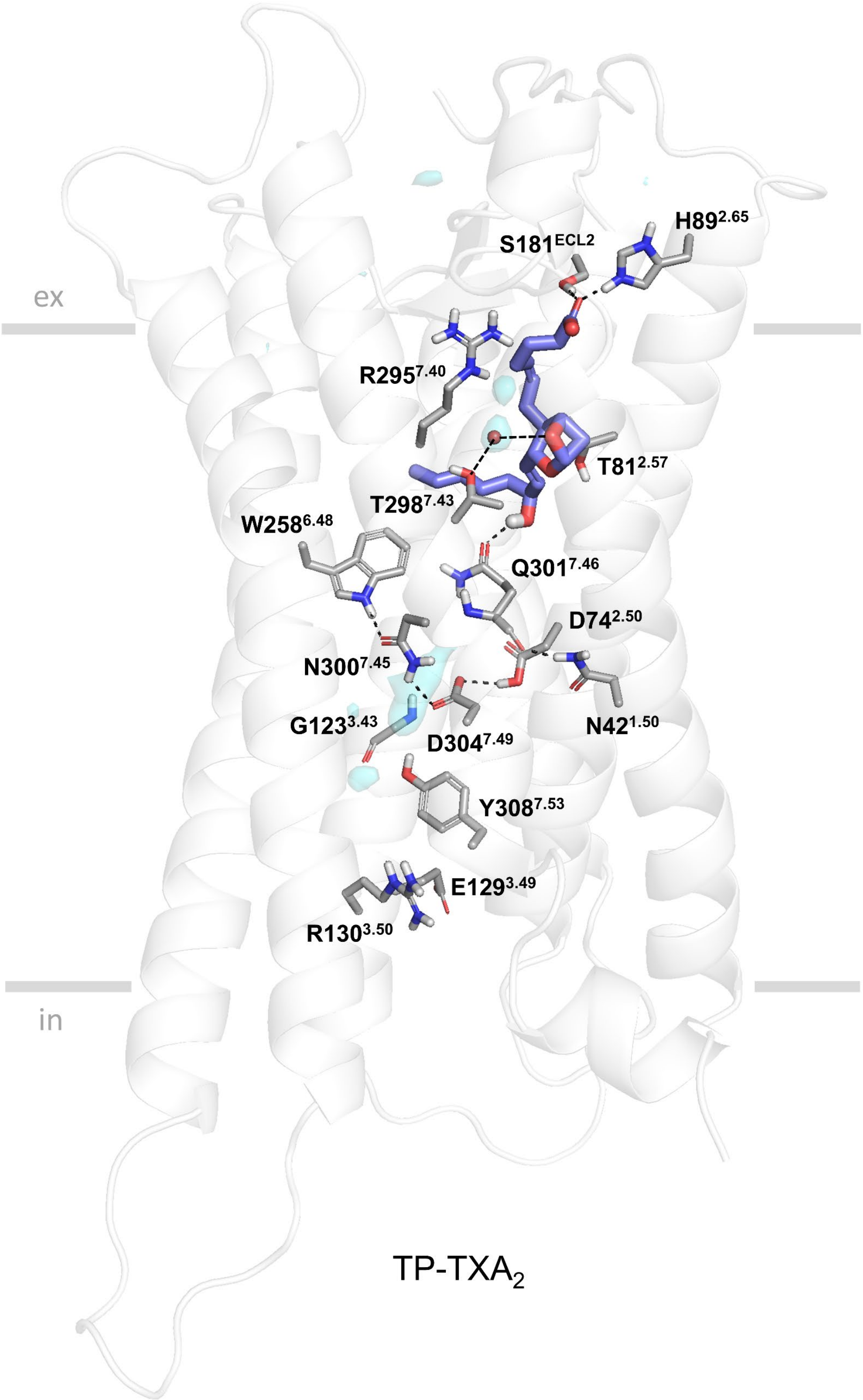

Supplementary Fig. 7 | Binding mode of TXA_2 and the TP from MD simulations. Representative snapshot of the TP bound to its endogenous ligand TXA_2 (violet) from MD simulations. Residues involved in a hydrogen bond network with the ligands and the activation mechanism are shown in stick representation. Hydrogen bonds are shown as black-dashed lines. Water clusters observed in the MD simulations are shown in cyan surface representation. A water molecule, which facilitates interaction between the bicyclic ring oxygen of the agonists and T298$^{7.43}$, is depicted as a red ball representing the oxygen atom.

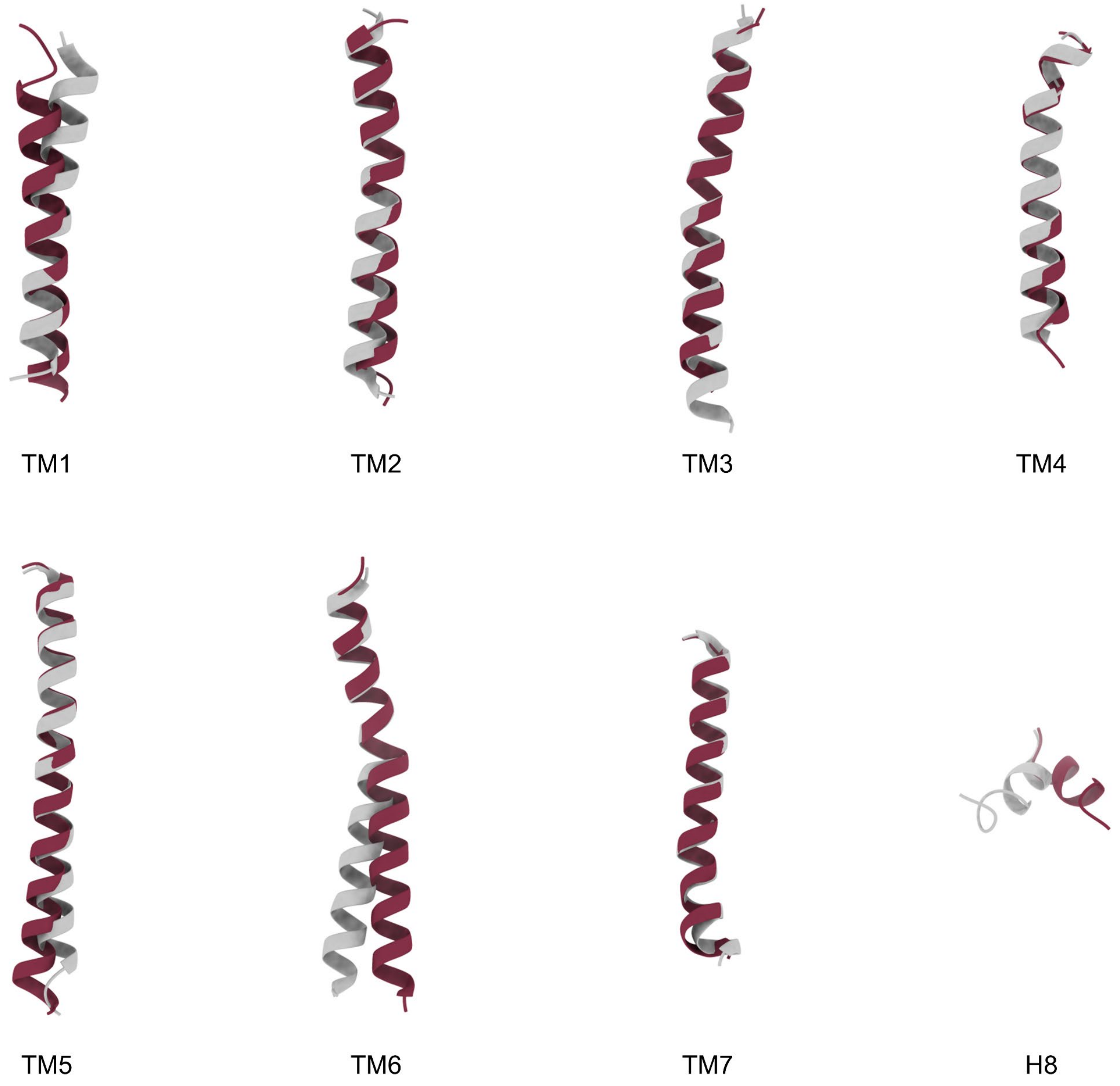

Supplementary Fig. 8 | Comparison of active and inactive states of the TP by helix. Structural alignment of transmembrane and C-terminal helix positions in the active state receptor bound to U46619 (red) and inactive structure bound to ramatroban (PDB ID 6IIU, grey), showing pronounced conformational changes at the extracellular (upper) face of TM1, and at the intracellular (lower) segment of TM6 and H8.

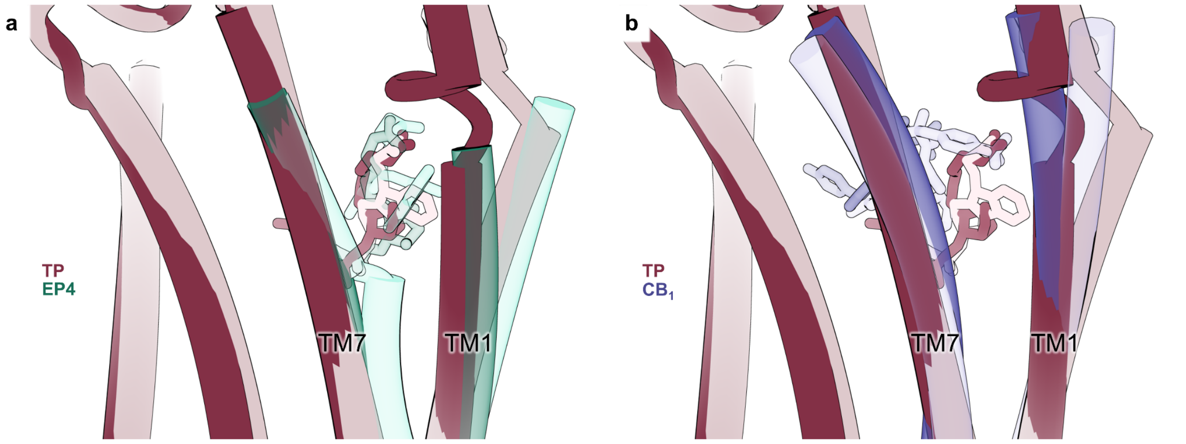

Supplementary Fig. 9 | Relative movement of TM1 and TM7 in the TP and other receptors upon activation. a Comparing the TP (red, active – U46619; pink, inactive – ramatroban) with EP4 (active – PGE_2, dark green – PDB 7D7M; inactive – ONO-AE3-208, light green – PDB 5YMY) indicating that the conformational change of TM1 associated with receptor activation is larger in the TP. **b** Comparing the TP (red, active – U46619; pink, inactive – ramatroban) with the cannabinoid receptor type 1 (CB_1) (purple, active – MDMB-fubinaca - PDB 6N4B; light purple, inactive – taranabant - PDB 5U09) showing a significantly larger opening of the gap in the TP. CB_1 is an example of a non-prostanoid receptor.

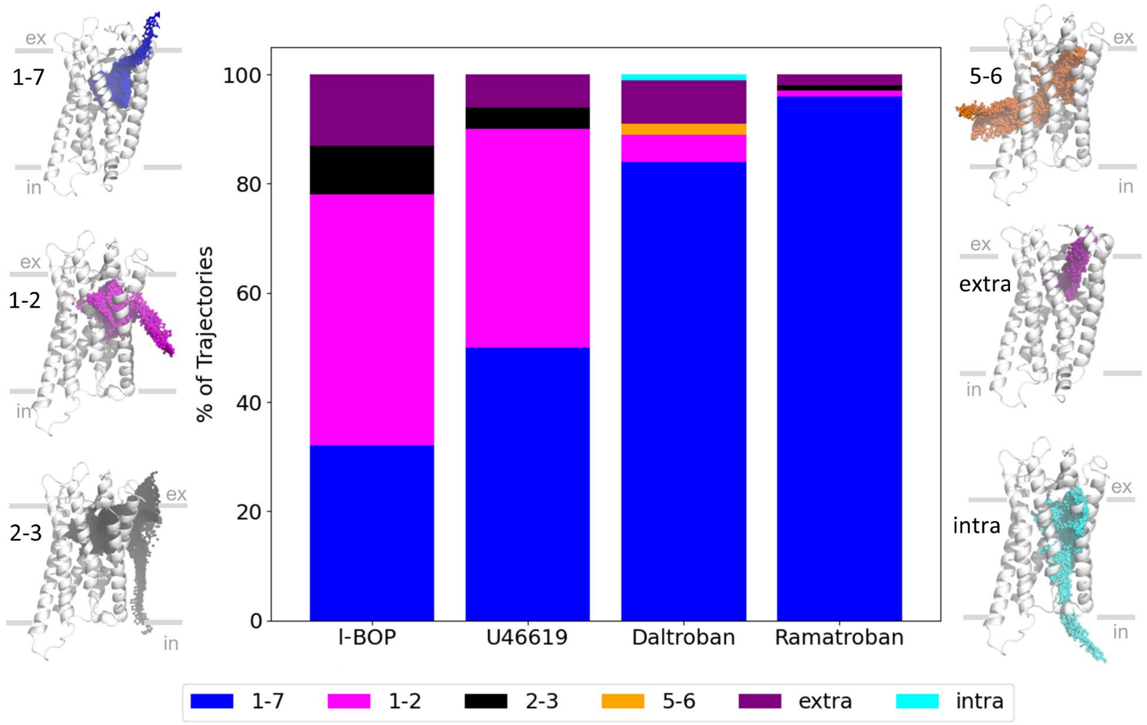

Supplementary Fig. 10 | Routes by which agonists and antagonists dissociate from the TP as determined by RAMD simulations. The bar plot shows the percentage of each ligand exiting route observed in RAMD simulations of the agonist and antagonist complexes. Dissociation routes lie between TM1 and TM7 (1-7, blue), between TM1 and TM2 (1-2, magenta), between TM2 and TM3 (2-3, black), between TM5 and TM6 (5-6, orange), towards the extracellular milieu (extra, purple), and towards the cytoplasm (intra, cyan). Percentage values are reported in the Supplementary Discussion.

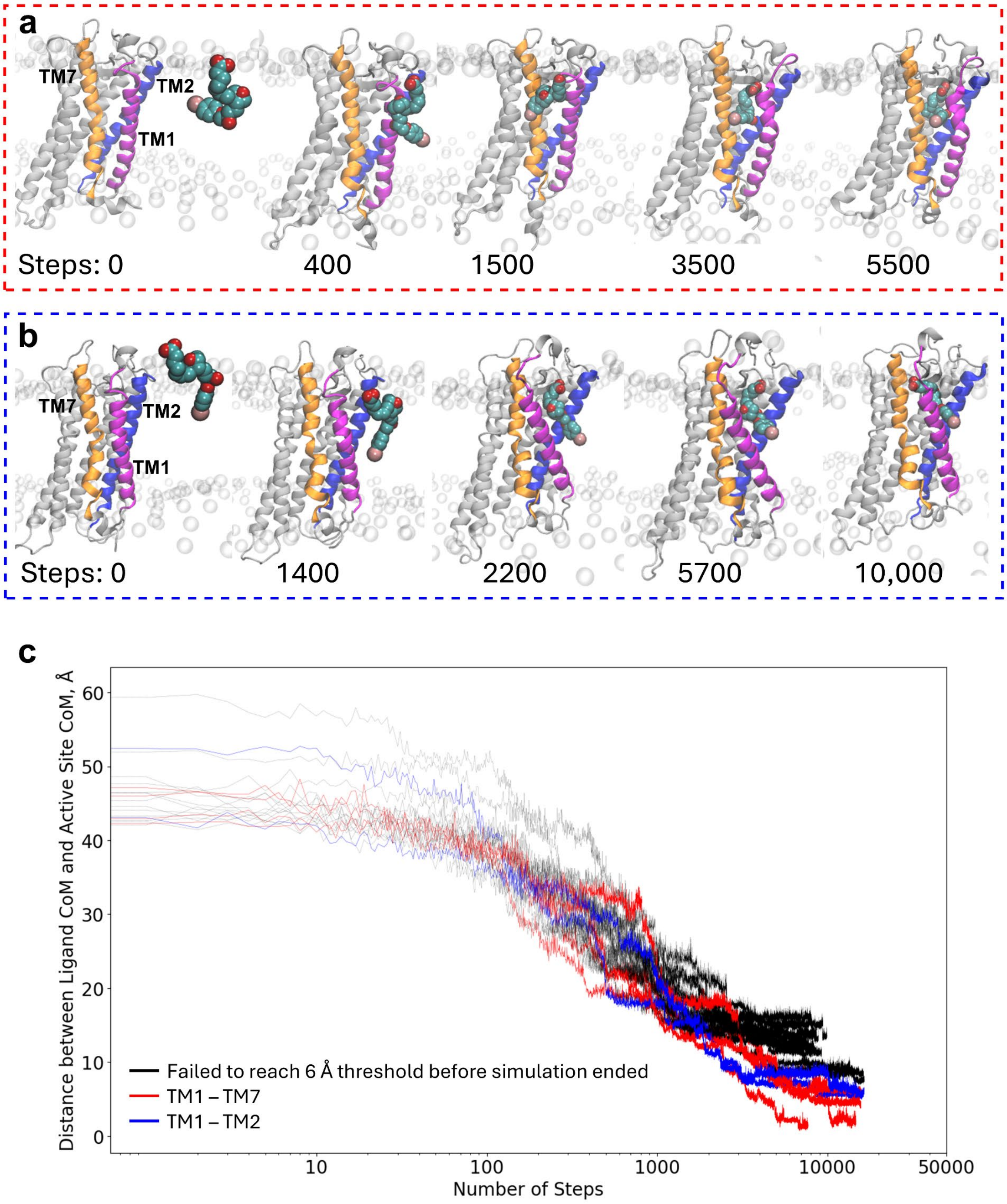

Supplementary Fig. 11 | I-BOP entry pathways through TM1-TM7 and TM1-TM2 gaps in the TP.
a-b suMD simulation snapshots showing I-BOP (teal carbon spheres) binding to the TP through two distinct membrane entry pathways: **(a)** via the TM1-TM7 gap and **(b)** via the TM1-TM2 gap. Transmembrane helices are colored as follows: TM1 (purple), TM2 (blue), and TM7 (orange). Numbers indicate simulation steps progressing from ligand approach to binding site entry. **c** Distance evolution between centers of mass (CoM) of I-BOP and the TP binding site across 20 independent suMD simulations. The x-axis represents the cumulative number of 150,000-step simulation cycles (both successful and unsuccessful attempts) performed for each system. The y-axis shows the distance between ligand and binding site CoMs. Six simulations successfully reached the 6 Å threshold: four trajectories (red) entered via the TM1-TM7 gap, with two of these achieving close approach (≤2 Å), while two trajectories (blue) entered via the TM1-TM2 gap. Simulations that did not reach 6 Å by the time the simulation ended are shown in black.

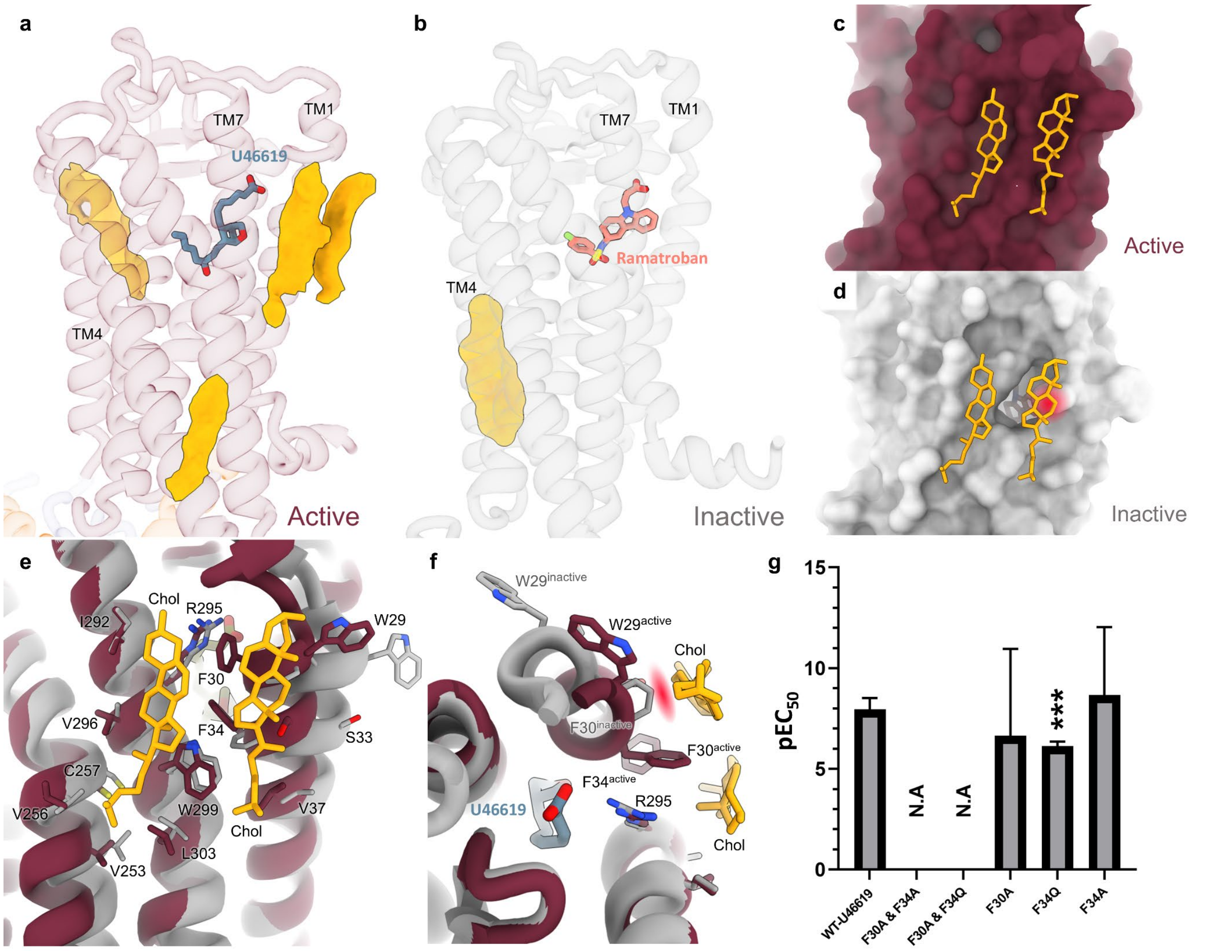

Supplementary Fig. 12 | Cholesterol binding at the TM1/7 cavity in the TP. a Four cholesterol* molecules were captured within the EM density. Three cholesterols are located toward the extracellular half of the receptor in close proximity to TM1, TM6, and TM7, while one is toward the intracellular half of the receptor next to TM5. **b** Cholesterol was also found in the inactive crystal structure of TP (PDB 6IIU). In contrast to the active state, only one cholesterol molecule is bound in the inactive structure. It is located toward the intracellular half of the receptor in close proximilty to TM4. **c** Surface representation of TP in the active state (U46619-bound) with cholesterol bound next to the hydrophobic cavity created by the gate at the TM1/TM7 interface. **d** Superposition of cholesterol binding pose in the active state (U46619-bound), with the inactive receptor structure (grey, PDB 6IIU) indicating that the cavity for cholesterol binding is absent in the inactive receptor state. In addition, the conformational change in F30$^{1.38}$ in the inactive state clashed with the cholesterol position in the active state. Clash is indicated in red. **e** Residues that compose the cholesterol gate binding site in the active TP (red) superposed with their couterparts in the inactive TP (grey, PDB 6IIU). **f** Top view of the superposition presented in (**e**), highlighting the clash (red) between F30$^{1.38}$ of the inactive state and one of the bound cholesterol molecules in the active state. **g** EC_{50} values in response to mutations in the residues composing the TP gap/cholesterol binding cavity. EC_{50} values are not available for mutants marked with N.A as their data did not fit dose-response curves. Data are presented as mean ± SEM from three independent experiments. *** indicates $P < 0.001$. Statistical significance was determined by one-way ANOVA followed by Dunnett's post-hoc tests, comparing the mutants treated with U46619 to the corresponding WT treated with U46619.

*Note that the TP was purified in the presence of cholesteryl hemisuccinate (CHS) that can hydrolyze to cholesterol. We thus modeled cholesterol, albeit we cannot exclude the possibility that some of the modeled cholesterol moieties could be CHS.

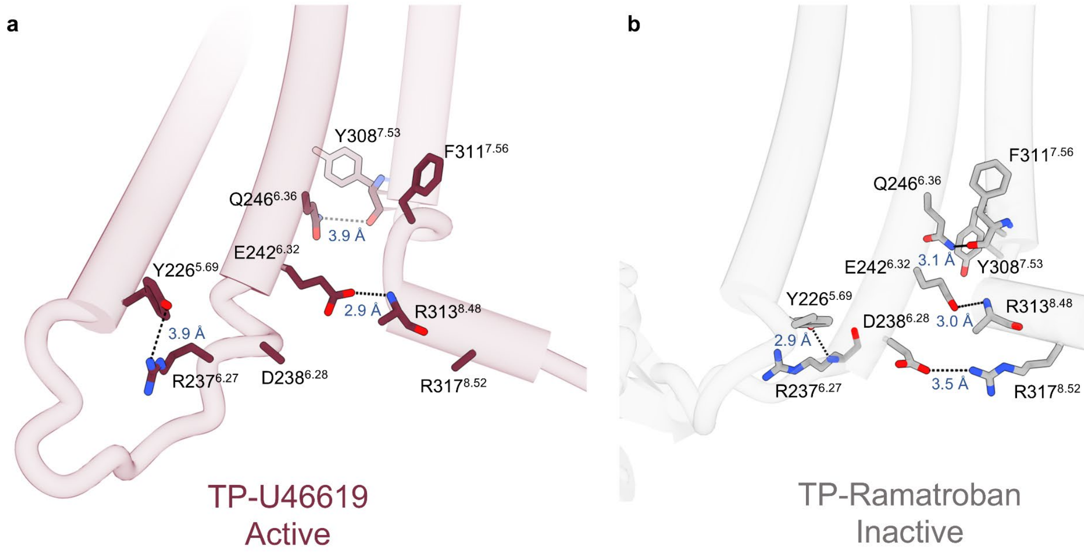

Supplementary Fig. 13 | Conformational differences between the intracellular face of the active and inactive states of the TP. a Hydrogen bond network among residues in the U46619-bound active TP. **b** Hydrogen bond network among residues in the inactive TP (ramatroban, PDB 6IIU). Hydrogen bonds are marked with a dashed line with distances in Å.

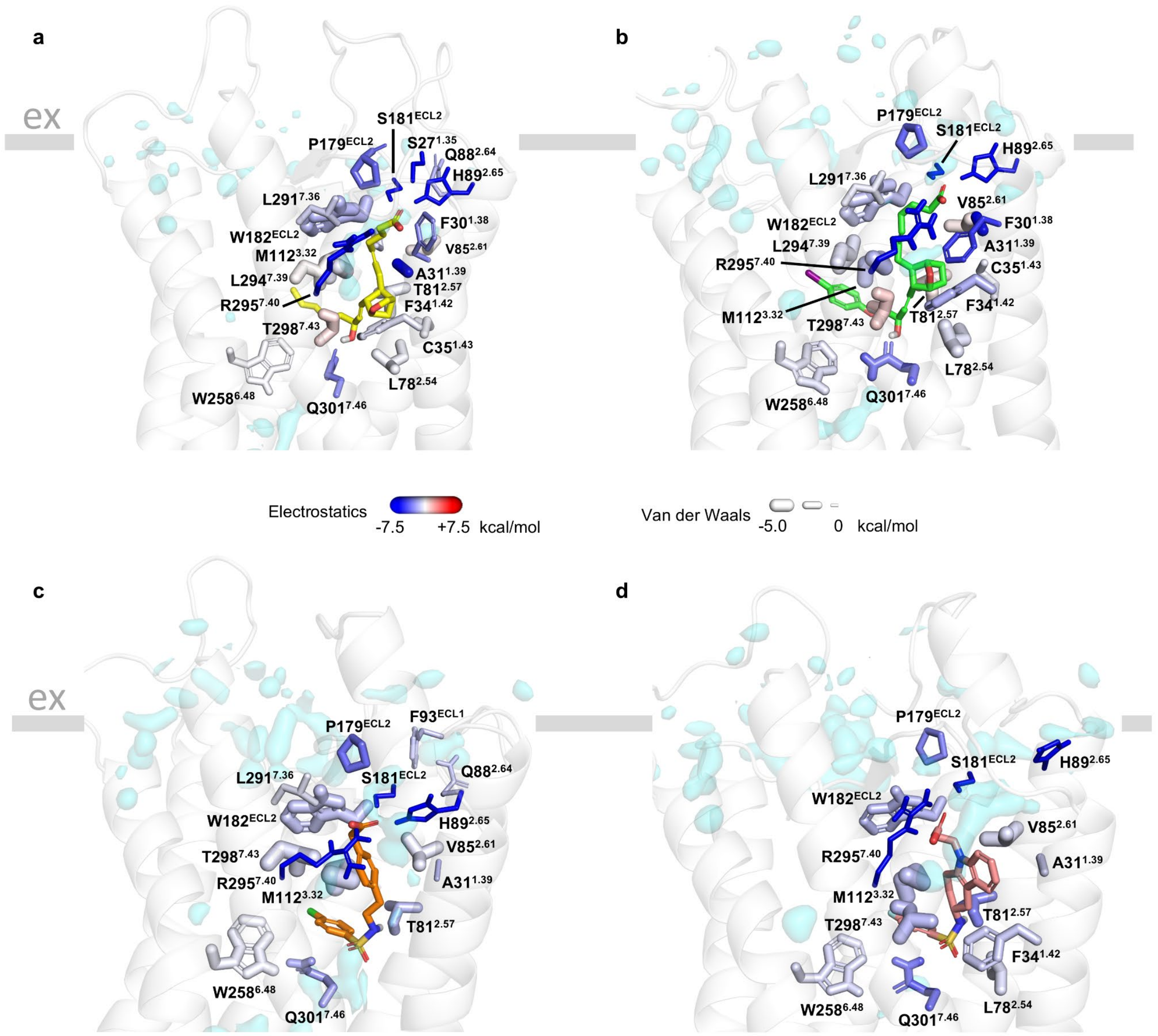

Supplementary Fig. 14 | Ligand-residue interactions in the TP probed by MD simulations. a TP-U46619. **b** TP-I-BOP. **c** TP-Daltroban. **d** TP-Ramatroban. A representative frame is shown with key residues forming contacts with U46619 (yellow), I-BOP (green), daltroban (orange), and ramatroban (pink). The size and colour of the residues correspond to the average strength of van der Waals and electrostatic interactions with the ligands, respectively (key as inset). Water clusters observed in the MD simulations are shown in cyan surface representation.

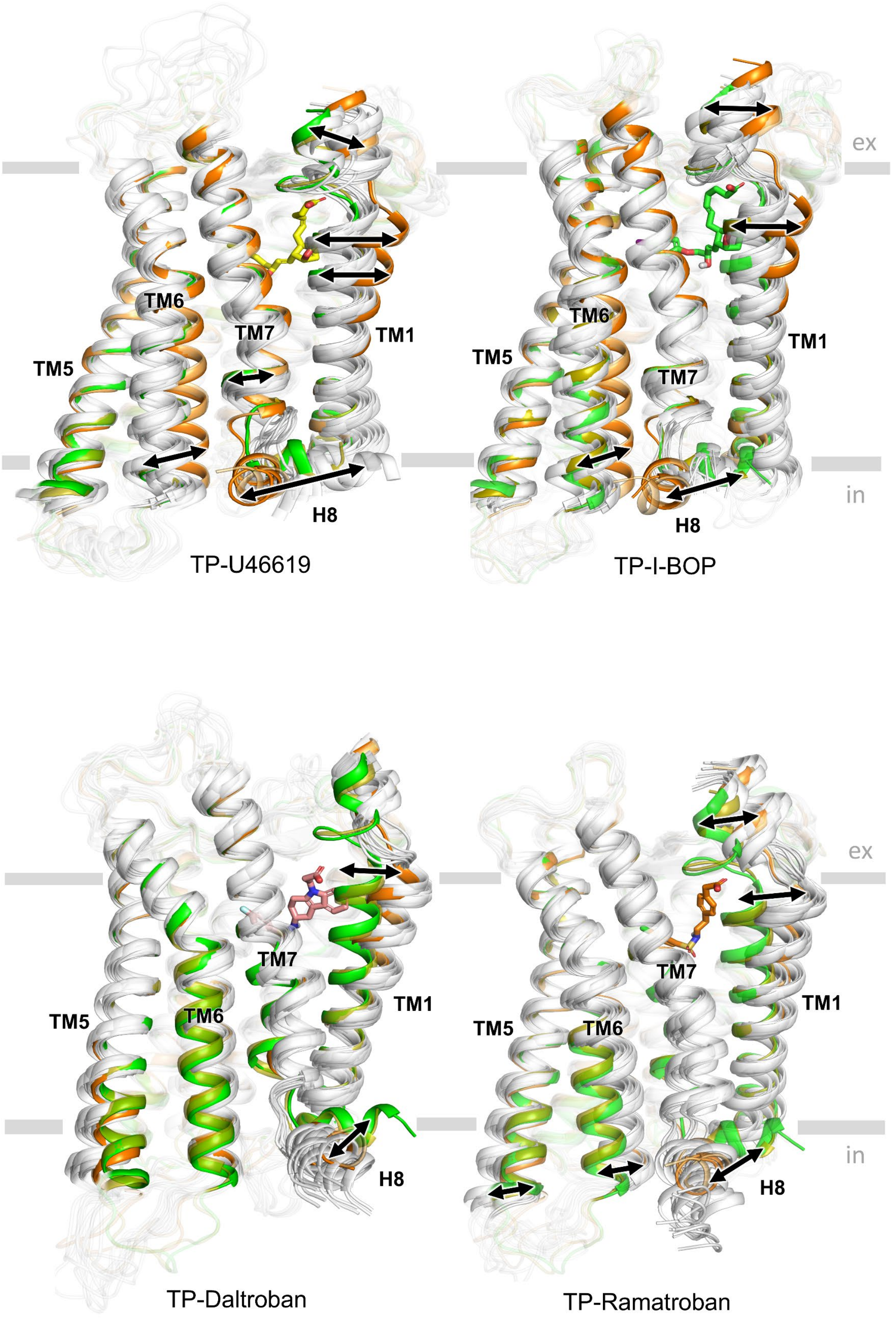

Supplementary Fig. 15 | Agonists and antagonists differentially affect transmembrane helix mobility in the TP. Overlays compare representative conformations from MD simulations of I-BOP-, U46619-, daltroban-, and ramatroban-bound receptor complexes (white) with corresponding experimental structures (light and normal green, light and normal orange, respectively). Arrows indicate the most notable fluctuations of TM1, TM5, TM6, TM7, and H8 between an active to inactive state during the simulations.

Supplementary Tables

Supplementary Table 1. Cryo-EM data collection, refinement, and validation statistics.

	TP-U46619 (EMDB-51324) (PDB 9GG5)	TP-I-BOP (EMDB-51324) (PDB 9GGG)
**Data collection and processing**		
Magnification	105,000	105,000
Voltage (kV)	300	300
Electron exposure (e–/Å^2)	38.4	38.4
Defocus range (μm)	-0.8-(-2.0)	-0.8-(-2.0)
Pixel size (Å)	0.826	0.826
Symmetry imposed	C1	C1
Initial particle images (no.)	5,425,932	3,512,010
Final particle images (no.)	216,383	242,712
Map resolution (Å)	3.26 (3.31,3.35)	3.25 (3.52,3.22)
FSC threshold	0.143	0.143
Map resolution range (Å)	3.26-12.59	3.25-12.74
**Refinement**		
Initial model used (PDB code)	6IIU	6IIU
Model resolution (Å)	3.6	3.6
FSC threshold	0.5	0.5
Model resolution range (Å)	3.26-12.59	3.25-12.74
Map sharpening *B* factor (Å^2)	121 (111.7,136.6)	123.9(135.0,125.2)
Model composition		
Non-hydrogen atoms	6,938	8,656
Protein residues	929	1159
Ligands	5	5
B factors (Å^2)		
Protein	92.04	181.81
Ligand	71.03	122.66
R.m.s. deviations		
Bond lengths (Å)	0.002	0.002
Bond angles (°)	0.423	0.420
Validation		
MolProbity score	1.15 (99th percentile)	1.25 (99th percentile)
Clashscore	1.69 (99th percentile)	1.83 (99th percentile)
Poor rotamers (%)	0	0
Ramachandran plot		
Favored (%)	96.51	95.63
Allowed (%)	3.38	4.37
Disallowed (%)	0.11	0

*Numbers in parentheses are of the locally refined receptor and G_q heterotrimer, respectively.

Supplementary Table 2. The average RMSD and RMSF of the receptor systems. RMSD and RMSF values were calculated for the Cα atoms of the entire receptor, the 7-transmembrane helical bundle (7TM) Cα atoms, and the ligand (non-hydrogen atoms). The calculations involved three 1 µs MD simulations for each receptor system. RMSD values were first averaged within each replica and combined between three replicas to yield the mean and standard deviation. RMSF values are also given as mean ± standard deviation between three different replicates.

Receptor Systems*	RMSD, Å			RMSF, Å		
	Cα	7TM-Cα	Ligand	Cα	7TM-Cα	Ligand
TP_{U46619}	3.1 ± 0.2	1.8 ± 0.1	1.8 ± 0.1	1.2 ± 0.0	0.8 ± 0.0	1.0 ± 0.1
TP_{empty_U46619}	2.8 ± 0.2	1.6 ± 0.1		1.3 ± 0.0	0.8 ± 0.1	
$TP_{U46619\_H89\_unprot}$	3.2 ± 0.3	2.1 ± 0.2	1.9 ± 0.5	1.4 ± 0.0	1.0 ± 0.0	1.4 ± 0.0
$TP_{I\text{-}BOP}$	2.6 ± 0.4	1.7 ± 0.3	2.0 ± 0.3	1.2 ± 0.1	0.9 ± 0.1	1.1 ± 0.1
$TP_{empty\_I\text{-}BOP}$	2.7 ± 0.2	1.7 ± 0.1		1.1 ± 0.1	0.8 ± 0.1	
$TP_{Ramatroban}$	2.6 ± 0.3	1.7 ± 0.3	1.6 ± 0.3	1.1 ± 0.1	0.8 ± 0.1	1.1 ± 0.2
$TP_{empty\_Ramatroban}$	2.7 ± 0.2	1.9 ± 0.2		1.2 ± 0.1	0.9 ± 0.1	
$TP_{Daltroban}$	2.7 ± 0.3	1.7 ± 0.1	2.0 ± 0.1	1.1 ± 0.2	0.8 ± 0.1	1.1 ± 0.0
$TP_{empty\_Daltroban}$	2.6 ± 0.1	1.6 ± 0.2		1.1 ± 0.1	0.8 ± 0.1	

*Receptor systems include the TP-U46619 complex (TP_{U46619}), TP-I-BOP complex ($TP_{I\text{-}BOP}$), TP-Ramatroban complex ($TP_{Ramatroban}$), TP-Daltroban complex ($TP_{Daltroban}$), and the TP-U46619, TP-I-BOP, TP-Ramatroban, and TP-Daltroban complexes from which the ligand has been removed (TP_{empty_U46619}, $TP_{empty\_I\text{-}BOP}$, $TP_{empty\_\_Ramatroban}$, and $TP_{empty\_\_Daltroban}$).

Supplementary Table 3. MD simulations reliability and reproducibility checklist.

Question	Yes	No	Response
**1. Convergence of simulations and analysis**			
1a. Is an evaluation presented in the text to show that the property being measured has equilibrated in the simulations (*e.g.* time-course analysis)?	+		The Methods subsections "Molecular dynamics simulations", "Ligand escape", "Agonist binding", and "Evaluation of H89 protonation state" include information about equilibration procedures.
1b. Then, is it described in the text how simulations are split into equilibration and production runs and how much data were analysed from production runs?	+		The Methods subsections "Molecular dynamics simulations", "Ligand escape", "Agonist binding", and "Evaluation of H89 protonation state" include information about how the simulations were split.
1c. Are there at least 3 simulations per simulation condition with statistical analysis?	+		Classical simulations have at least three replicas. Constant pH simulations use from 3-6 simulations per pH value to calculate the converged values for the local pK_a's. τ-RAMD simulations have 100 repeats per ligand. 20 repeats were carried out for SuMD association trajectories.
1d. Is evidence provided in the text that the simulation results presented are independent of initial configuration?	+		Production runs were started from slightly different initial geometries.
**2. Connection to experiments**			
2a. Are calculations provided that can connect to experiments (*e.g.* loss or gain in function from mutagenesis, binding assays, NMR chemical shifts, J-couplings, SAXS curves, interaction distances or FRET distances, structure factors, diffusion coefficients, bulk modulus and other mechanical properties, *etc.*)?	+		Classical, association, and dissociation simulation results are discussed in relation to mutagenesis and cryo-EM data.
**3. Method choice**			
3a. Do simulations contain membranes, membrane proteins, intrinsically disordered proteins, glycans, nucleic acids, polymers, or cryptic ligand binding?	+		Simulations contain membranes and membrane proteins. Details are provided in the Methods section.

3b. Is it described in the text whether the accuracy of the chosen model(s) is sufficient to address the question(s) under investigation (e.g. all-atom vs. coarse-grained models, fixed charge vs. polarizable force fields, implicit vs. explicit solvent or membrane, force field and water model, etc.)?	+		
3c. Is the timescale of the event(s) under investigation beyond the brute-force MD simulation timescale in this study that enhanced sampling methods are needed?	+		
If **YES**, are the parameters and convergence criteria for the enhanced sampling method clearly stated?	+		For association trajectories obtained using SuMD, the criteria to stop is based on the distance between binding site residues and the ligand CoM. The threshold distance is provided in the Methods subsection “Agonist binding”.
If **NO,** is the evidence provided in the text?	☐	☐	
**4. Code and reproducibility**			
4a. Is a table provided describing the system setup that includes simulation box dimensions, total number of atoms, total number of water molecules, salt concentration, lipid composition (number of molecules and type)?		+	All this information is provided in the Methods section as text rather than a table.
4b. Is it described in the text what simulation and analysis software and which versions are used?	+		Software and their versions are provided in the Methods section.
4c. Are other parameters for the system setup described in the text, such as protonation state, type of structural restraints if applied, nonbonded cutoff, thermostat and barostat, etc.?	+		Protonation state for H89 was decided based on constant pH simulations. Unless default parameters are used, thermostat and barostat types were mentioned in the Methods section.
4d. Are initial coordinate and simulation input files and a coordinate file of the final output provided as supplementary files or in a public repository?	+		Initial coordinates and input files with an example association trajectory are provided in Zenodo (https://doi.org/10.5281/zenodo.17848900).

4e. Is there custom code or custom force field parameters?		+		
	If **YES**, are they provided as supplementary files or in a public repository?	+		The original SuMD script was adjusted so that it could be applied for systems larger than 100000 atoms. It is provided in Zenodo (https://doi.org/10.5281/zenodo.17848900).

Supplementary References

1. Kurz M, Krett AL, Bunemann M. Voltage Dependence of Prostanoid Receptors. *Mol Pharmacol* **97**, 267-277 (2020).
2. Tao X, Zhao C, MacKinnon R. Membrane protein isolation and structure determination in cell-derived membrane vesicles. *Proc Natl Acad Sci U S A* **120**, e2302325120 (2023).
3. Kirchhofer SB*, et al.* Differential interaction patterns of opioid analgesics with micro opioid receptors correlate with ligand-specific voltage sensitivity. *Elife* **12**, (2023).
4. Punjani A, Rubinstein JL, Fleet DJ, Brubaker MA. cryoSPARC: algorithms for rapid unsupervised cryo-EM structure determination. *Nature methods* **14**, 290-296 (2017).
5. Punjani A, Zhang H, Fleet DJ. Non-uniform refinement: adaptive regularization improves single-particle cryo-EM reconstruction. *Nature methods* **17**, 1214-1221 (2020).
6. Afonine PV*, et al.* Real-space refinement in PHENIX for cryo-EM and crystallography. *Acta Crystallogr D Struct Biol* **74**, 531-544 (2018).
7. Kimanius D, Dong L, Sharov G, Nakane T, Scheres SHW. New tools for automated cryo-EM single-particle analysis in RELION-4.0. *Biochem J* **478**, 4169-4185 (2021).
