# Supplementary material for: Structural and dynamic insights into agonist recognition and function of the thromboxane A2 receptor
Source: Nat Commun. 2026 Feb 23;17:3071. doi: 10.1038/s41467-026-69844-9 (PMC13039517; doi:10.1038/s41467-026-69844-9)
Supplement: Supplementary file 2 — Reporting Summary [file 41467_2026_69844_MOESM2_ESM.pdf]

nature portfolio

Corresponding author(s): Martin Caffrey, Irina Tikhonova, and Moran Shalev-Benami

Last updated by author(s): Dec 21, 2025

# Reporting Summary

Nature Portfolio wishes to improve the reproducibility of the work that we publish. This form provides structure for consistency and transparency in reporting. For further information on Nature Portfolio policies, see our Editorial Policies and the Editorial Policy Checklist.

## Statistics

For all statistical analyses, confirm that the following items are present in the figure legend, table legend, main text, or Methods section.

| n/a | Confirmed | |
|---|---|---|
| ☐ | ☒ | The exact sample size (*n*) for each experimental group/condition, given as a discrete number and unit of measurement |
| ☐ | ☒ | A statement on whether measurements were taken from distinct samples or whether the same sample was measured repeatedly |
| ☐ | ☒ | The statistical test(s) used AND whether they are one- or two-sided<br>*Only common tests should be described solely by name; describe more complex techniques in the Methods section.* |
| ☐ | ☒ | A description of all covariates tested |
| ☐ | ☒ | A description of any assumptions or corrections, such as tests of normality and adjustment for multiple comparisons |
| ☐ | ☒ | A full description of the statistical parameters including central tendency (e.g. means) or other basic estimates (e.g. regression coefficient) AND variation (e.g. standard deviation) or associated estimates of uncertainty (e.g. confidence intervals) |
| ☐ | ☒ | For null hypothesis testing, the test statistic (e.g. *F*, *t*, *r*) with confidence intervals, effect sizes, degrees of freedom and *P* value noted<br>*Give P values as exact values whenever suitable.* |
| ☒ | ☐ | For Bayesian analysis, information on the choice of priors and Markov chain Monte Carlo settings |
| ☒ | ☐ | For hierarchical and complex designs, identification of the appropriate level for tests and full reporting of outcomes |
| ☒ | ☐ | Estimates of effect sizes (e.g. Cohen's *d*, Pearson's *r*), indicating how they were calculated |

*Our web collection on statistics for biologists contains articles on many of the points above.*

## Software and code

Policy information about availability of computer code

| | |
|---|---|
| Data collection | EPU 3.6 software for cryo-EM data collection. |
| Data analysis | cryoSPARC v4.2.1, Relion 4.0, Phenix 1.20.1, Coot 0.9.8.93, ChimeraX 1.8, GraphPad Prism 10, Schrodinger Maestro 2021-3, Amber20, MDAnalysis 2.7.0, NAMD2, VMD 1.9.3, Glide (from Maestro), and GROMACS 2020.5. |

For manuscripts utilizing custom algorithms or software that are central to the research but not yet described in published literature, software must be made available to editors and reviewers. We strongly encourage code deposition in a community repository (e.g. GitHub). See the Nature Portfolio guidelines for submitting code & software for further information.

## Data

Policy information about availability of data

All manuscripts must include a data availability statement. This statement should provide the following information, where applicable:

- Accession codes, unique identifiers, or web links for publicly available datasets
- A description of any restrictions on data availability
- For clinical datasets or third party data, please ensure that the statement adheres to our policy

All data needed to evaluate the conclusions in the paper are present in the paper and/or the Supplementary Materials. Structures in the paper have been deposited in the Protein Data Bank (PDB) under accession codes 9GG5 [https://doi.org/10.2210/pdb9GG5/pdb] (TP-U46619); and 9GGG [https://doi.org/10.2210/pdb9GGG/pdb] (TP-I-BOP). The cryo-EM electrostatic potential maps have been deposited in the Electron Microscopy Data Bank (EMDB) under accession codes EMD-51324 [https://www.ebi.ac.uk/pdbe/entry/emdb/EMD-51324] (TP-U46619); and EMD-51331 [https://www.ebi.ac.uk/pdbe/entry/emdb/EMD-51331] (TP-I-BOP). The

following previously reported structures were used for structure analysis: 6IIU [https://doi.org/10.2210/pdb6IIU/pdb] (TP-ramatroban); 6IIV [https://doi.org/10.2210/pdb6IIV/pdb] (TP-daltroban); 7D7M [https://doi.org/10.2210/pdb7D7M/pdb] (EP4-PGE2); 5YWY [https://doi.org/10.2210/pdb5YWY/pdb] (EP4-ONO-AE3-208); 6N4B [https://doi.org/10.2210/pdb6N4B/pdb] (CB1- MDMB-fubinaca); and 5U09 [https://doi.org/10.2210/pdb5U09/pdb] (CB1- taranabant). The source data for the MD simulation protocols are available at this link: https://doi.org/10.5281/zenodo.17848900.

## Research involving human participants, their data, or biological material

Policy information about studies with human participants or human data. See also policy information about sex, gender (identity/presentation), and sexual orientation and race, ethnicity and racism.

| | |
|---|---|
| Reporting on sex and gender | This information was not collected as our study did not involve human participants, data, or biological material. |
| Reporting on race, ethnicity, or other socially relevant groupings | *Please specify the socially constructed or socially relevant categorization variable(s) used in your manuscript and explain why they were used. Please note that such variables should not be used as proxies for other socially constructed/relevant variables (for example, race or ethnicity should not be used as a proxy for socioeconomic status).*<br>*Provide clear definitions of the relevant terms used, how they were provided (by the participants/respondents, the researchers, or third parties), and the method(s) used to classify people into the different categories (e.g. self-report, census or administrative data, social media data, etc.)*<br>*Please provide details about how you controlled for confounding variables in your analyses.* |
| Population characteristics | *Describe the covariate-relevant population characteristics of the human research participants (e.g. age, genotypic information, past and current diagnosis and treatment categories). If you filled out the behavioural & social sciences study design questions and have nothing to add here, write "See above."* |
| Recruitment | *Describe how participants were recruited. Outline any potential self-selection bias or other biases that may be present and how these are likely to impact results.* |
| Ethics oversight | *Identify the organization(s) that approved the study protocol.* |

Note that full information on the approval of the study protocol must also be provided in the manuscript.

# Field-specific reporting

Please select the one below that is the best fit for your research. If you are not sure, read the appropriate sections before making your selection.

☒ Life sciences ☐ Behavioural & social sciences ☐ Ecological, evolutionary & environmental sciences

For a reference copy of the document with all sections, see nature.com/documents/nr-reporting-summary-flat.pdf

# Life sciences study design

All studies must disclose on these points even when the disclosure is negative.

| | |
|---|---|
| Sample size | No statistical method was used to predetermine the sample size. For BRET activity assays, at least three independent experiments (n=3) were performed, consistent with established practice in the literature. |
| Data exclusions | For BRET activity assays, data was only excluded from technical errors.<br><br>For cryo-EM single-particle analyses, data were excluded as part of the normal processing workflow. Micrographs were excluded based on poor CTF fit, thick ice, or contamination. Particles from junk classes were removed. |
| Replication | Protein purifications were replicated a number of times (>3) during protocol optimization.<br><br>BRET activity assays were performed in technical triplicate.<br><br>Classical MD simulations were repeated at least three times. Constant pH simulations used 3-6 simulations per pH value to calculate the converged values for the local pKa's. For τ-RAMD simulations, 100 repeats were performed per ligand. For SuMD association trajectories, 20 repeats were carried out. |
| Randomization | The only randomization in our study came from initial particle picking during cryo-EM data processing due to reference-free particle picking. |
| Blinding | Blinding was not applicable to any part of our study. |

# Reporting for specific materials, systems and methods

We require information from authors about some types of materials, experimental systems and methods used in many studies. Here, indicate whether each material, system or method listed is relevant to your study. If you are not sure if a list item applies to your research, read the appropriate section before selecting a response.

## Materials & experimental systems

| n/a | Involved in the study |
|---|---|
| ☒ | ☐ Antibodies |
| ☐ | ☒ Eukaryotic cell lines |
| ☒ | ☐ Palaeontology and archaeology |
| ☒ | ☐ Animals and other organisms |
| ☒ | ☐ Clinical data |
| ☒ | ☐ Dual use research of concern |
| ☒ | ☐ Plants |

## Methods

| n/a | Involved in the study |
|---|---|
| ☒ | ☐ ChIP-seq |
| ☒ | ☐ Flow cytometry |
| ☒ | ☐ MRI-based neuroimaging |

# Eukaryotic cell lines

Policy information about cell lines and Sex and Gender in Research

| | |
|---|---|
| Cell line source(s) | Spodoptera frugiperda (Sf9) insect cell line<br>Purchased from Oxford Expression Technologies. Catalogue number: 600100<br><br>Trichoplusia ni (Tni) Hi5 insect cell line<br>Purchased from Expression Systems. Catalogue number: 94-002F<br><br>Human Embryonic Kidney (HEK293) cell line<br>Purchased from ATCC. Catalogue number: CRL-1573 |
| Authentication | The cell lines were not authenticated after purchase. |
| Mycoplasma contamination | The Sf9 and Tni cell lines were not tested for mycoplasma contamination. HEK293 cells were regularly tested using the MycoAlert Plus detection kit (Lonza). |
| Commonly misidentified lines<br>(See ICLAC register) | No commonly misidentified cell lines were used. |

# Plants

| | |
|---|---|
| Seed stocks | *Report on the source of all seed stocks or other plant material used. If applicable, state the seed stock centre and catalogue number. If plant specimens were collected from the field, describe the collection location, date and sampling procedures.* |
| Novel plant genotypes | *Describe the methods by which all novel plant genotypes were produced. This includes those generated by transgenic approaches, gene editing, chemical/radiation-based mutagenesis and hybridization. For transgenic lines, describe the transformation method, the number of independent lines analyzed and the generation upon which experiments were performed. For gene-edited lines, describe the editor used, the endogenous sequence targeted for editing, the targeting guide RNA sequence (if applicable) and how the editor was applied.* |
| Authentication | *Describe any authentication procedures for each seed stock used or novel genotype generated. Describe any experiments used to assess the effect of a mutation and, where applicable, how potential secondary effects (e.g. second site T-DNA insertions, mosiacism, off-target gene editing) were examined.* |
